# High-speed and high-precision PbSe/PbI_2_ solution process mid-infrared camera

**DOI:** 10.1038/s41598-020-80847-4

**Published:** 2021-01-15

**Authors:** Hannaneh Dortaj, Mahboubeh Dolatyari, Armin Zarghami, Farid Alidoust, Ali Rostami, Samiye Matloub, Reza Yadipour

**Affiliations:** 1 Photonics and Nanocrystals Research Lab (PNRL), University of Tabriz, 5166614761 Tabriz, Iran; 2 SP-EPT Lab., ASEPE Company, Industrial Park of Advanced Technologies, 5364196795 Tabriz, Iran; 3 Quantum Photonics Research Lab (QPRL), University of Tabriz, 5166614761 Tabriz, Iran; 4 Faculty of Electrical and Computer Engineering, University of Tabriz, 5166614761 Tabriz, Iran

**Keywords:** Engineering, Nanoscience and technology, Optics and photonics

## Abstract

Infrared (IR) cameras based on semiconductors grown by epitaxial methods face two main challenges, which are cost and operating at room temperature. The alternative new technologies which can tackle these two difficulties develop new and facile material and methods. Moreover, the implementation of high speed camera, which makes high resolution images with normal methods, is very expensive. In this paper, a new nanostructure based on a cost-effective solution processed technology for the implementation of the high-speed mid-infrared light camera at room temperature is proposed. To this end, the chemically synthesized PbSe–PbI_2_ core–shell Quantum Dots (QDs) are used. In this work, a camera including 10 × 10 pixels is fabricated and synthesized QDs spin-coated on interdigitated contact (IDC) and then the fabricated system passivated by epoxy resin. Finally, using an electronic reading circuit, all pixels are converted to an image on the monitor. To model the fabricated camera, we solved Schrodinger–Poisson equations self consistently. Then output current from each pixel is modeled based on semiconductor physics and dark and photocurrent, as well as Responsivity and Detectivity, are calculated. Then the fabricated device is examined, and dark and photocurrents are measured and compared to the theoretical results. The obtained results indicate that the obtained theoretical and measured experimental results are in good agreement together. The fabricated detector is high speed with a rise time of 100 ns. With this speed, we can get 10 million frames per second; this means we can get very high-resolution images. The speed of operation is examined experimentally using a chopper that modulates input light with 50, 100, 250, and 500 Hz. It is shown that the fabricated device operates well in these situations, and it is not limited by the speed of detector. Finally, for the demonstration of the proposed device operation, some pictures and movies taken by the camera are attached and inserted in the paper.

## Introduction

Night Vision technology provides the ability to see in darkness, and a long way has been passed in the development of this technology from the 1950s. It is initially developed for military purposes and also used for many other applications such as law enforcement, hunting, surveillance, security, navigation, and hidden object detection^1^. Night vision technology basically works on the Infrared (IR) spectrum^2^. Thus, for monitoring objects in dark conditions, the infrared spectrum must be detected. It is common to apply the 3–5 µm infrared window for military uses, 8–12 µm window for thermal imaging and military uses, and > 20 µm for THz uses such as medical diagnostics^3^.

Night vision cameras mostly work in two main ways: Image enhancement and Thermal imaging. Image enhancement: First night vision devices built on this technology were developed during the Second World War^4^. The imaging mechanism is called image enhancement, which is based on the image intensifier tube. It is an electro-optical device which consists of three main components, a photocathode, a Microchannel Plate (MCP), and a phosphor screen, which amplifies the ambient light to achieve better vision^5–7^. Thermal imaging: This technology operates by detecting the far-infrared spectrum emitted as heat by objects^8^. All objects emit infrared energy as a function of their temperature^9^. So, a phased array of infrared detector elements is used to obtain the temperature information and translate into electric impulses that are sent to the display for creating an image^1,7^.

In this paper, a new method of imaging is introduced to design a mid-infrared camera based on the photodetector array structure by considering the absorption process of nanoparticles. Two main types of photodetectors are called as the photodiodes and the photoconductors. Photodiodes are formed by a junction between two different semiconductors. Under the influence of the built-in electrical field in the junction, electron–hole pairs are transmitted toward respective contacts and generate an electrical current^10^. On the other hand, a photoconductor typically consists of a piece of semiconductor material with metal contacts. By absorbing incident light, mobility or carrier density in the semiconductor changes, and subsequently, the conductivity of the material will change that causes an alteration in output electrical signal^11^. Following this concept, by using this type of photodetectors for the proposed camera, the output electric signals are sent to a signal-processing unit that transforms information from the photodetectors into data for the display.

Many experiments have been performed to find an appropriate material for detecting the infrared spectra, which requires a small-bandgap (*Eg* = 0.1 eV). However, growing, processing, and fabricating into other devices for such small-bandgap semiconductors are more difficult than large-bandgap ones^12^. Therefore, because of critical disadvantages, including growth-related difficulties, the cryogenic cooling requirement in mid and long infrared spectral ranges^13^, high-cost manufacturing and also for improving the detecting parameters at room temperature^3^, a lot of effort has done to develop photodetector structures from bulk to the quantum-based photodetectors, particularly quantum dot photodetectors^14^. In the QD structures, 3-D confinement has provided some interesting properties, including higher absorption coefficient, lower dark current, narrower spectral width absorption, higher photoconductive gain, size-dependent detectivity, and low-temperature processing^15–18^. So, because of the these advantages, QD photodetectors are preferred in many applications at room temperature, and also increasing the effective light detection area per each pixel makes them suitable for imaging devices^19^.

High-speed and high-sensitive infrared photodetectors are demanded for several applications such as night vision, optical communications and, short-wave infrared imaging^20–22^. Solution-processing simplifies ready integration with many varieties of substrates upon other integrated circuits^23^. Furthermore, the optical absorption and emission spectra of the quantum dots can widely be controlled via the quantum size effect^24^ to achieve tunable emission and absorption, which made them desirable for next-generation of high-speed infrared photodetectors^25,26^. Solution-processed materials such as colloidal quantum dots provide higher absorption, low-cost manufacturing, room-temperature processing, and ease of large-area fabrication on rigid or flexible substrates^27,28^, which have obviously proposed as a candidate material for optoelectronic devices like photodetectors^29,30^.

## Methods and materials

### Synthesis of PbSe/PbI2 nanoparticles

PbI_2_ (461 mg, 1 mmol) were dissolved in oleylamine (OLA) (1 mL) and 5 g trioctylphosphine oxide (TOPO) in a 250 mL three-necked flask under nitrogen atmosphere at 200 °C. 79 mg (1.0 mmol) of selenium was dissolved in 5 mL melted triphenylphosphine (PPh_3_) and injected at a temperature of 200 °C into this flask. The temperature was stabilized at 110 °C for 2 h during the growth of the particles. The resulting precipitate was re-dispersed in n-hexane and centrifuged, then washed several times sequentially with n-hexane, ethanol, and 2-propanol and finally dried at 40 °C for 2 h^31^. The obtained powders were dispersed in 2-propanol, and KI (280 mg, 1.7 mmol) was added to the solution and stirred for 24 h at room temperature. The obtained particles were washed several times with water, ethanol, and acetone till no OLA, TOPO, or PPh_3_ is found in the washing medium (checked by 1H and 31P NMR) and finally dried at 40 °C for 2 h^31^.

### Fabrication of detectors

0.01 g of the synthesized *PbSe/PbI*_2_ particles were dispersed in 2-propanol and spin coated on interdigitated 10 × 10 array made by copper contacts deposited on a fiberglass substrate. At the end, the fabricated system was passived by epoxy resin. For this purpose, 1 g epoxy resine mixed with 0.5 g hardner and poured on the device. The ambient parameters of the system include: the temperature is 300 K, and the incident optical power is 10 µW/cm^2^. The energy of photons of the monochromatic IR light, which radiates on the photodetector, is 0*.*354 eV, so its wavelength is equal to 3.5 µm that is suitably designed at the range of mid-infrared. For absorbing UV and Visible and near infrared photons we used mounted color filter.

## Simulation and discussion

### Structure description

**Figure 1:**
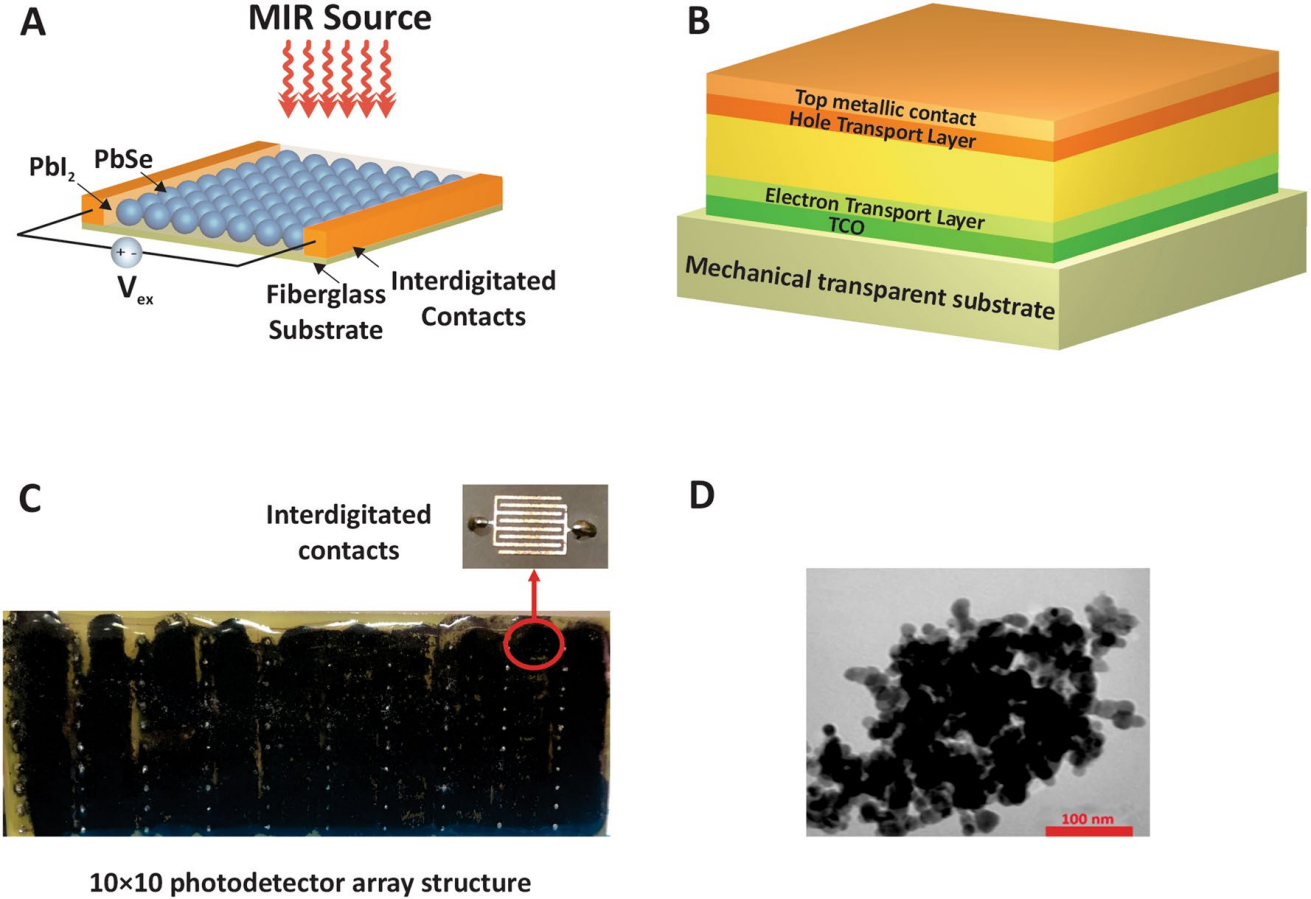
Schematic illustration of MIR-to-visible light upconversion device. Structure of the analyzed infrared detectors in (**A**) planar and (**B**) vertical geometry model^32^. (**C**) Real image of the fabricated photodetector array structure. (**D**) TEM image of synthesized PbSe/PbI_2_ nanoparticles^31^.

Two models have been designed for the fabrication of infrared detectors, which are planar and vertical geometry model (see Fig. 1A,B). The planar model is cost-effective to fabrication, and it is not sensitive to the film quality, and roughness and cracks don’t effect on electrical properties of the device. Interdigitated electrodes are used for enhancing the current magnitude. The film of nanoparticles is coated on the substrate by the methods like spin coating, drop-casting, dip coating, or spray coating^32^. The second method is based on a vertical geometry method. In this method, the nanoparticles deposited on transparent electrodes like ITO or FTO. However, this method is expensive with low sensitivity, and we preferred to use the first method for the fabrication of the mid-infrared camera (3–5 µm). The proposed system consists of a 10 × 10 array of interdigitated contacts with the active region placed on them to form a photodetector array structure.

Among all criteria to build an IR detector, selecting nanomaterial as an absorber is important. Between all nanoparticles used as an absorber in photodetectors, lead chalcogenides are the best solution (because of their sensitivity) for this purpose. In this regard, PbS is suitable for short infrared wavelengths, and PbSe acts as a perfect candidate for mid-infrared wavelengths^33–38^. In our case, the active region is covered by synthesized PbSe QDs. The dimensions of length, width, and thickness in the active region are considered as 6 mm, 3 mm, and 1 mm, respectively. For fabricating the photodetector, the synthesized and surface modified PbSe particles were dispersed in 2-propanol and spin coated on interdigitated copper contacts deposited on a fiberglass substrate^31^. A schematic and real image of the fabricated photodetector array structure is shown in Fig. 1A,C.

**Figure 2:**
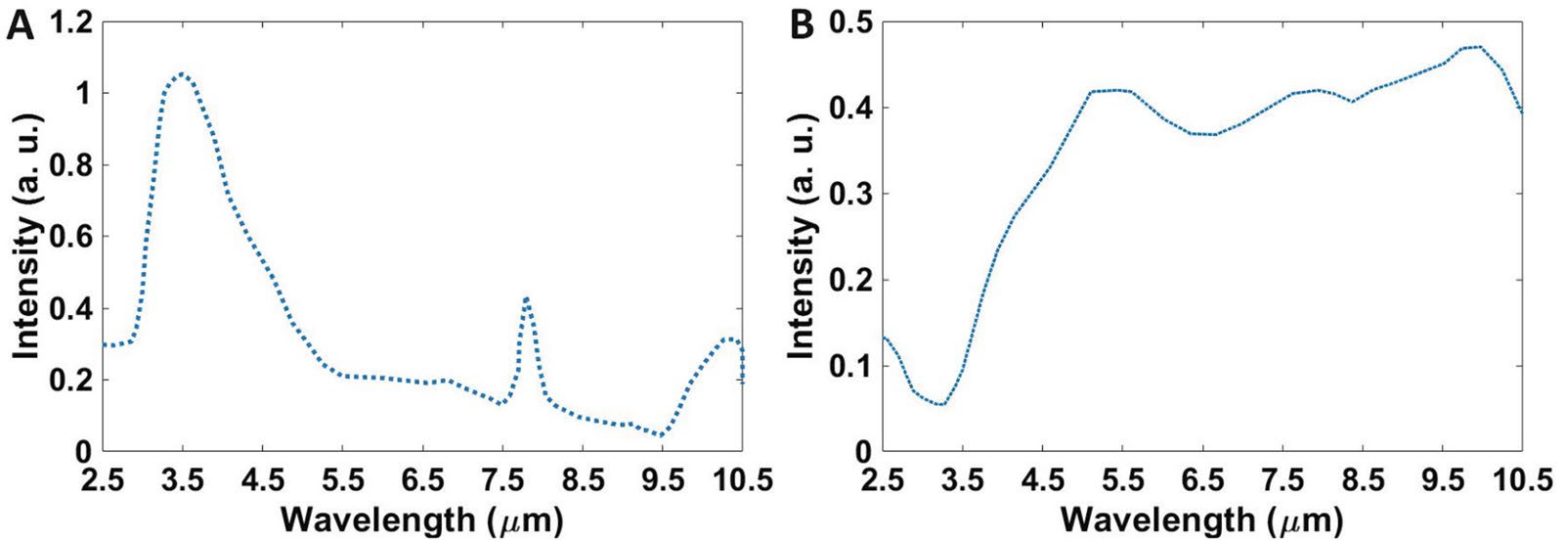
Absorption spectra of (**A**) PbSe, (**B**) epoxy resin.

**Figure 3:**
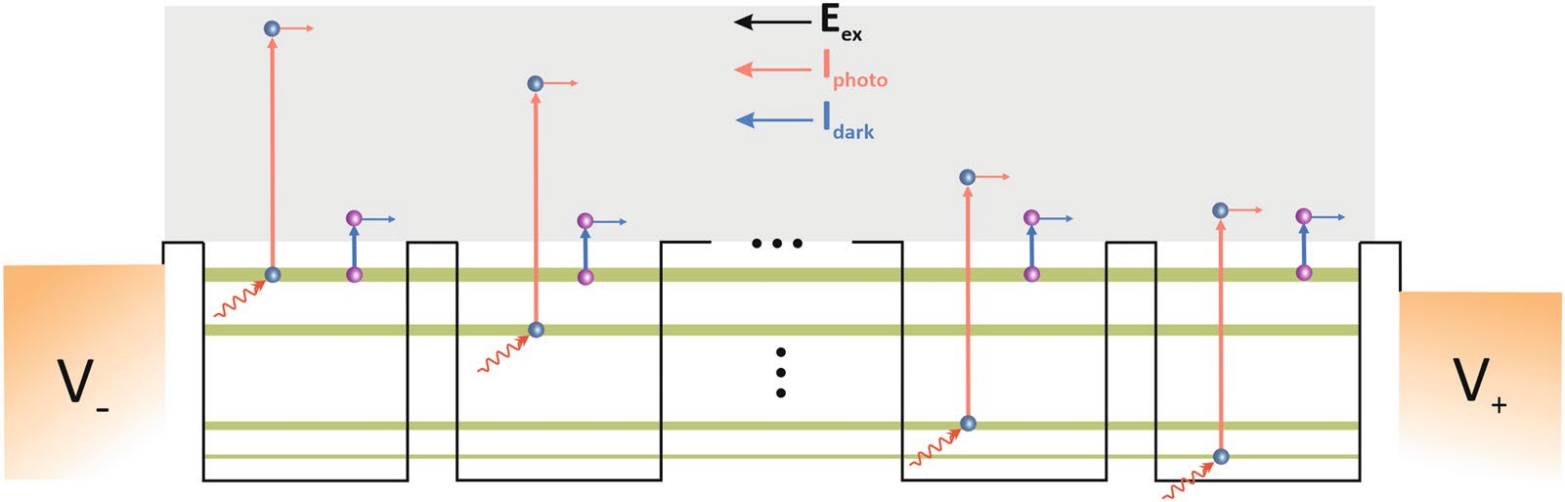
Mechanism of generating the output current through the intersubband absorption process. Flowing in the continuum band, E_ex_ is labeled for external electrical field, dark current is labeled as I_dark_ which is generating thermally and photo current is labeled as I_photo_ which is generating optically.

Regarding the geometrical features, in the introduced structure, the radius of dots are presumed uniform and spherical. As the TEM image of synthesized material shows, the diameter of spherical QDs is 30 nm^31^ Fig. 1D. As in the previous researches has been reported^39–43^, the surface engineering of nanoparticles affects the physical, especially electronic properties of them and in photodetectors, engineering of the trap states is possible in this way. As a result of our previous works, we reached a fast and sensitive detector using engineering of the surface of PbSe QDs by I^−^ ions^31^. In this work, we exchanged the long-chain oleate ions with iodine ions to get a very sensitive camera without speed limits because of the used QDs. In this condition, at the surface of *PbSe* QDs, we have *PbI*_2_, and we can calculate the *PbSe/PbI*_2_ core/shell system as the infrared absorber. The difference between the affinities of core and shell in defined QDs is about 0.3 eV, so the difference between the sub-band states and the edge of the continuous band is at least 0*.*3 eV, which is suitable for absorption in the mid-IR (3–5 µm) spectrum. Figure 2 shows absorption spectra of PbSe and epoxy resin. As figure shows the absorption band of nanoparticles located at 3–5 µm. Epoxy resin (which has been used as passivation material for device) has absorption in this area too and it can reduce sensitivity of the detector. However, we didn’t have suitable case for this purpose and we used that for passivation of device. The absorption mechanism is shown in Fig. 3. In this figure, intersubband to continuum band transitions are considered.

### Modeling

**Figure 4:**
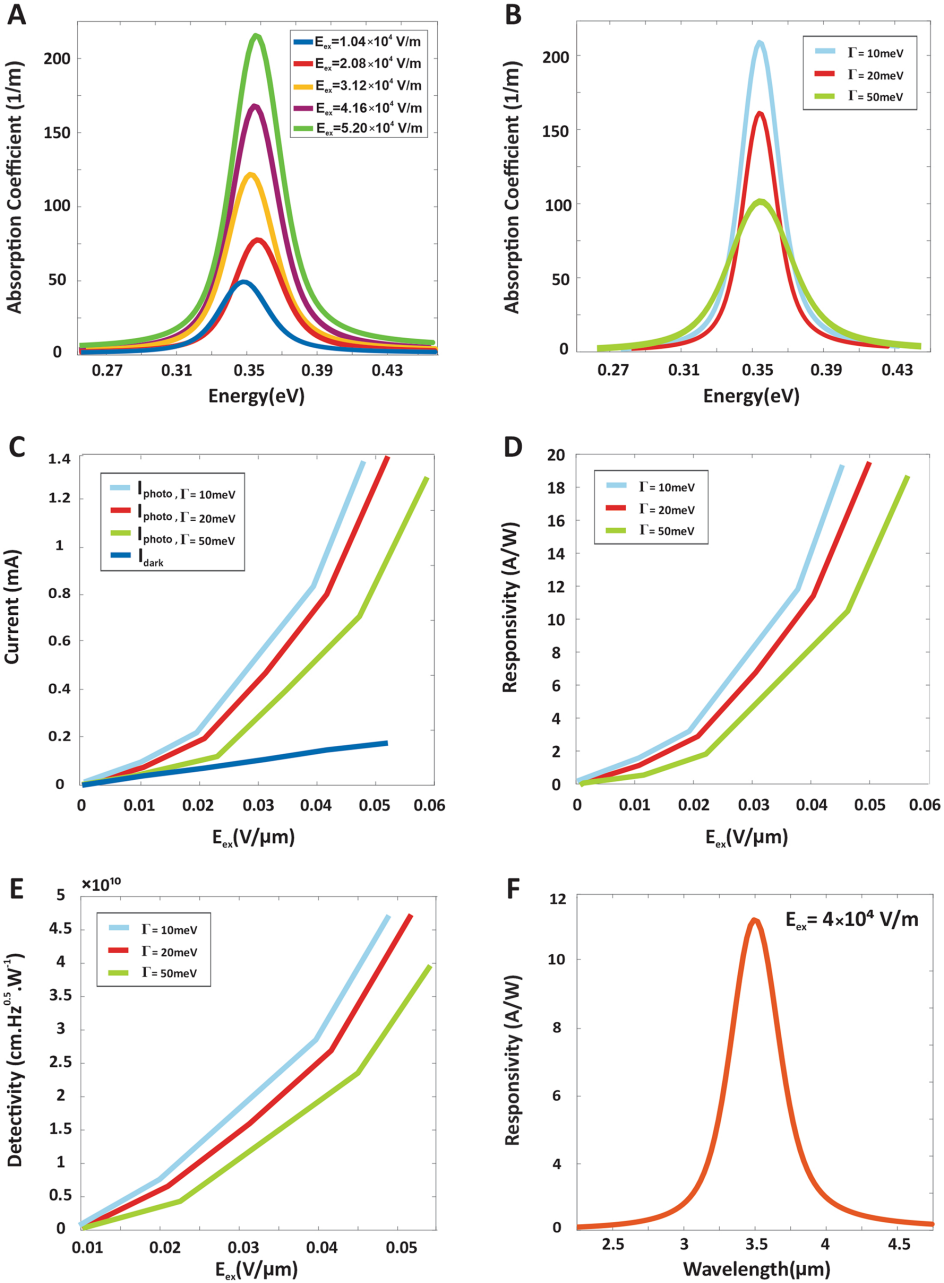
The photodetector’s performance parameters simulated by FEA software considering the inhomogeneous broadening with ${\Gamma } = 10\,{\text{meV}}, 20{\text{meV \,and }}50\,{\text{meV}}.$ (**A**) Absorption coefficient under different energies (The peak is in $\hbar$ *ω* = 0.354 eV, λ = 3.5 µm), (**B**) absorption coefficient for different ${\Gamma }$s. (**C**) Dark current and photocurrent, (**D**) responsivity, (**E**) Detectivity of the defined system versus the applied external field and (**F**) responsivity for mid-IR spectrum.

In the proposed structure, the external bias is applied through the interdigitated contacts, which we have assumed as a constant external potential. The electrons can contribute to the output electrical current in two ways. Without illumination, the dark current is generated thermally, and the photocurrent is generated optically by absorbing the incident IR light. The output current produced through these two mechanisms can flow in the continuum band, which is proportional to the intensity of incident light. In this structure, the tunneling process wasn’t considered, because it was much less than other components of the device’s dark current. By using a Finite Element Analysis software, the photodetector’s performance parameters are computed. To begin through Eq. (1)^44,45^ the defined system’s dark current has been calculated in different applied fields:

1 $$ I_{dark} = ev_{d} n_{total} A,{ } $$

where *e* is the electron charge, *v*_*d*_ is the average electron drift velocity, *n*_*total*_ is the electrons concentration excited out of the quantum dots by the thermionic emission and tunneling^46^, and *A* is the photosensitive area of the photodetector. Here *v*_*d*_ is:

2 $$ v_{d} = \frac{{\mu F_{ex} }}{{\sqrt {1 + \left( {\frac{{\mu F_{ex} }}{{v_{s} }}} \right)^{2} } }}, $$

in which *µ* is the electron’s mobility, *F*_*ex*_ is the external electric field, and *v*_*s*_ is the saturated velocity. The photocurrent in photoconductive detectors such as introduced structure is dependent on the quantum efficiency and the incident number of photons^42^ that is achieved by:

3 $$ I_{ph} = \eta eA\varphi g_{ph} , $$

in which *η* is the quantum efficiency, *φ* is the photon flux density (defined as the ratio of the incident optical power to the incident photon energy), and *g*_*ph*_ is the photoconductive gain. Quantum efficiency, the ratio of electrons exciting from the sub-band states to the continuum states to the incident number of photons, is obtained through^47^:

4 $$ \eta = 1 - e^{ - \alpha h} , $$

where *α* is the absorption coefficient for intersubband transitions, and *h* is the thickness of the deposited material. So, in order to obtain the photocurrent, the structure absorption coefficient for the intersubband transitions should be calculated by using Fermi’s golden rule^48^ through Eq. (5). For this purpose, the absorption coefficient of all the transitions whose peaks are in the specified energy (0*.*354 eV*, γ* = 0*.*02 eV) was calculated and summed up. The peak of the absorption coefficient in the desired wavelength (3.5 µm) at different external electric fields is shown in Fig. 4A.

5 $$\alpha \left( {h\omega } \right) = \left( {\frac{\omega }{{n_{r} c\varepsilon_{0} }}} \right)\mathop \sum \limits_{f} \mathop \sum \limits_{i} \frac{{\left| {\mu_{if} } \right|^{2} \gamma }}{{\left( {E_{f} - E_{i} - h\omega } \right)^{2} + \gamma^{2} }} \left( {N_{i} - N_{f} } \right)G\left( {E - E_{fi} } \right), $$

6 $$ G\left( {E - E_{fi} } \right) = \frac{1}{{\sqrt {2\pi } \xi_{0} }}exp\left[ { - \frac{{\left( {E - E_{fi} } \right)^{2} }}{{}}2\xi_{0}^{2} } \right]. $$

In which $\hbar$ is the reduced Plank constant, *ω* is the optical angular frequency ($\hbar$*ω* is the incident photon energy), *n*_*r*_ is the refractive index of the material, *c* is the speed of the light in free space, *s*_0_ is the permittivity of the free space^49^, *µ*_*if*_ is the intersubband optical dipole matrix element between initial and final states, 2*γ* is the line width broadening which has been considered for homogenous broadening arising from carrier scattering process, *i* is the index of the sub-band states (initial states), *f* is the index of the continuum states (final states), *N*_*i*(*f*)_ illustrates the number of electrons per unit volume in the i_th_ (*f*_th_) sub-band (continuum) states, and finally *G*(*E − E*_*fi*_) is defined as Gaussian distribution function attained by Eq. (6), that is applied due to the effects of QDs size non-uniformity on the absorption of the proposed structure^50^. Considering solution process technology, the size of each group of QDs relates to the desired wavelengths can be deviated from the central value of the radius give rise to the distribution of energy levels in each QD ensembles. The inhomogeneous broadening of energy levels can be modeled by the Gaussian function in which $\xi_{0}$ $\left( {FWHM = {\Gamma } = 2.35\xi_{0} } \right)$ is QD coverage^51–53^ and the effect of this model on simulation results are shown in Fig. 4B–E.

The photoconductive gain, the fraction of electrons excited by photons contributing in the output electrical current is governed by the ratio of the relaxation time *τ*_*life*_, and the transit time *τ*_*trans*_ (Eq. (7))^54,55^.

7 $$ g_{ph} = \frac{{\tau_{life} }}{{\tau_{trans} }}, $$

in which *τ*_*life*_ = 4*e*^*−*8^*, µ* = 1050 cm^2^/V s were assumed for *PbSe* quantum dots^56^. As we know, in the photoconductor devices in contrast to the photodiodes, the gain is higher than one due to the higher carrier lifetime. The faster decay under higher biases represents that the carriers are transmitted to the contacts under a high electric field. So, the dependency of transit time and applied bias is obtained through^26^:

8 $$ \tau_{trans} = \frac{{L^{2} }}{\mu V}, $$

in which *µ* is the electron mobility, *L* is the film thickness, and *V* is the applied bias voltage. Finally, with the quantum efficiency and photoconductive gain in hand, the given system’s photocurrent can be obtained through Eq. (3), and the results are presented in Fig. 4C. In the last step, after calculating the dark current and photocurrent of the device, in order to obtain the device’s performance and making it easier to compare the characteristics of different detectors, the specific detectivity should be calculated^57^. So first, the current responsivity of the device in the unit of *A/W* is attained through Eq. (9)^47^, and the results under different applied external biases are presented in Fig. 4D:

9 $$ R = \eta \frac{e}{h\omega }g_{ph} . $$

Next, the device’s noise current should be calculated. Different methods can participate in the noise current, including shot noise, Johnson noise, and 1*/f* noise. In a resistive device, such as the proposed photodetector, thermal or Johnson noise arising from the electrons thermal motion is evaluated by^54^:

10 $$ I_{n}^{2} = 4K_{B} TI_{dark} \Delta f, $$

in which *k*_*B*_ is the Boltzman constant, *T* is the temperature, ∆*f* is the noise bandwidth of an integrating filter. By calculating the device’s responsivity and noise current, the device’s detectivity can be obtained through^47^:

11 $$ D^{*} = \frac{{R\sqrt {A\Delta f} }}{{I_{n} }}. $$

In which *R* is the responsivity and *A* is the area of photodetector’s photosensitive region. The unit of the normalized detectivity is defined as ‘Jones’ (cm Hz/W) which expresses the sensitivity of the detector. Finally, the detectivity of the defined device is presented in Fig. 4E.

## Experimental results

**Figure 5:**
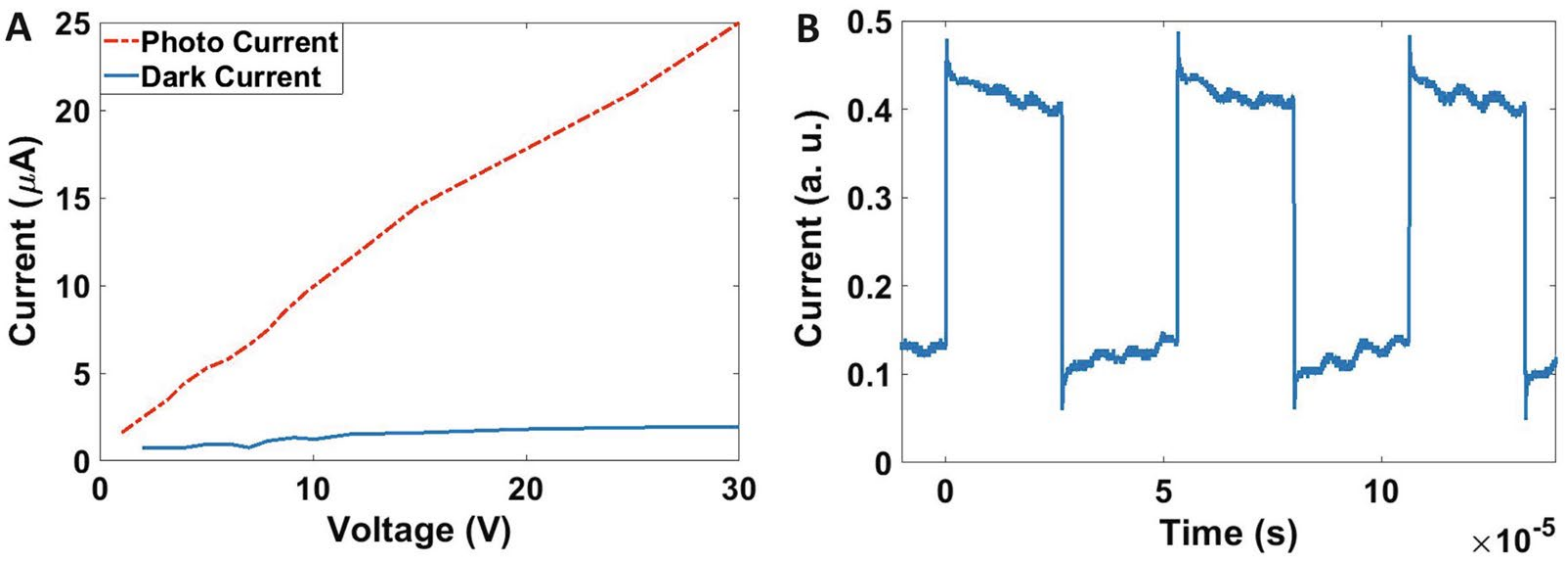
Experimental results for fabricated material. (**A**) The I–V and (**B**) response time of the detectors made by *PbSe/PbI*_2_ QDs.

**Figure 6:**
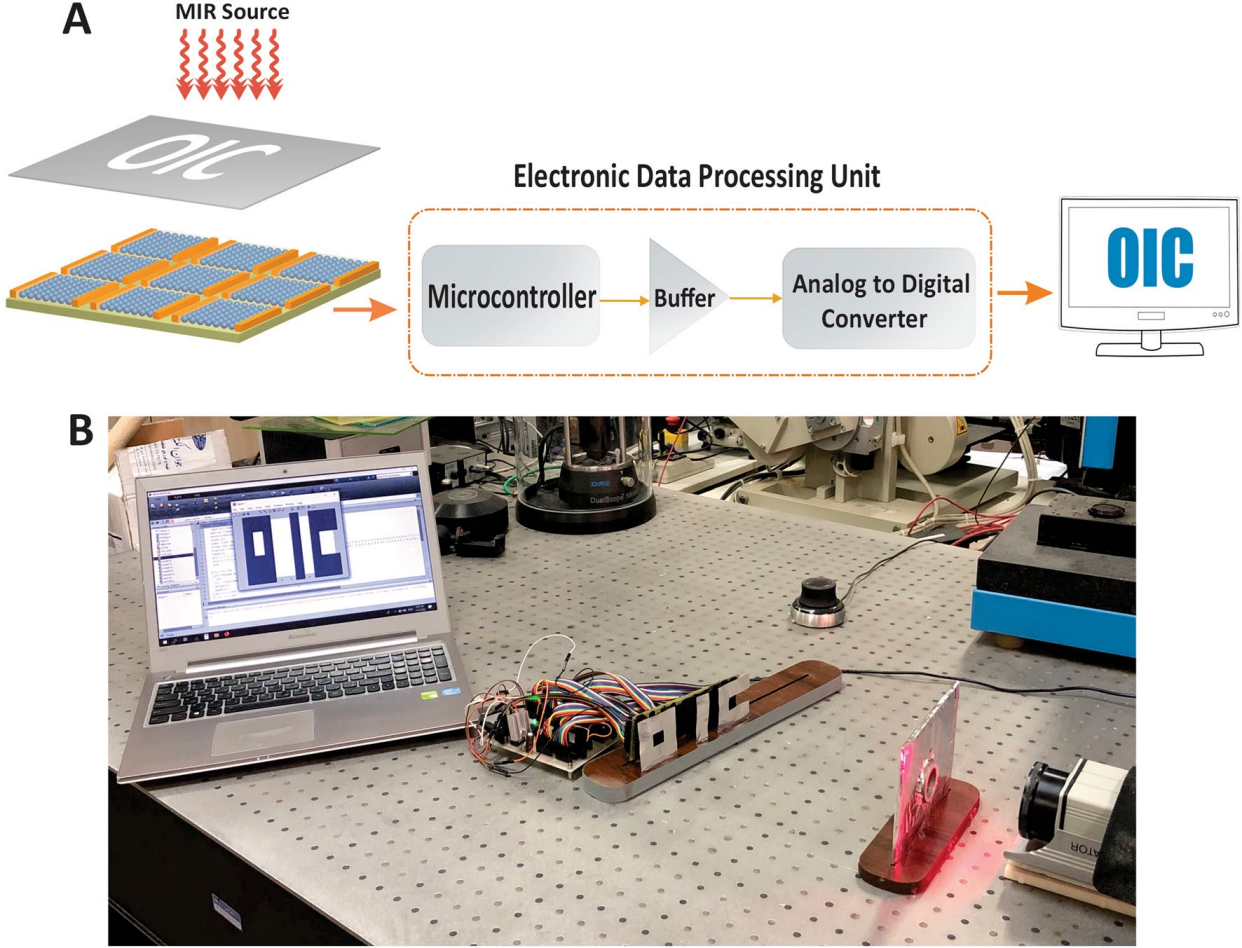
Imaging process by using the designed electronic circuit. (**A**) Block diagram of the electronic circuit for electrical signal-processing. (**B**) Real image of the fabricated IR camera.

The I–V and response time of the detectors made by *PbSe/PbI*_2_ QDs are presented in Fig. 5^31^. As the figure shows, the rise time of the detector is 100 ns. For recording an image, an electronic circuit for biasing the fabricated 10 × 10 array measuring their ohmic resistance, and processing output electric signal, was designed to display an image as shown in Fig. 6A. This electronic data processing unit consists of four blocks: in the first block, a microprocessor gets the resistances of 100 sensors. The second block is a buffer. Because the input resistance of the microprocessor can’t be ignored against the high resistances of photodetector sensors, a loading effect appears. So, a buffer Op-Amp is used for the impedance matching between the sensors’ resistances and the microprocessor’s input impedance and also for amplifying the flowing current. In the third block, an analog to digital converter is used to transform the values of the resistances via a serial port. Finally, in the last block, we can receive the data from the serial port and monitor them in an image at PC.

**Figure 7:**
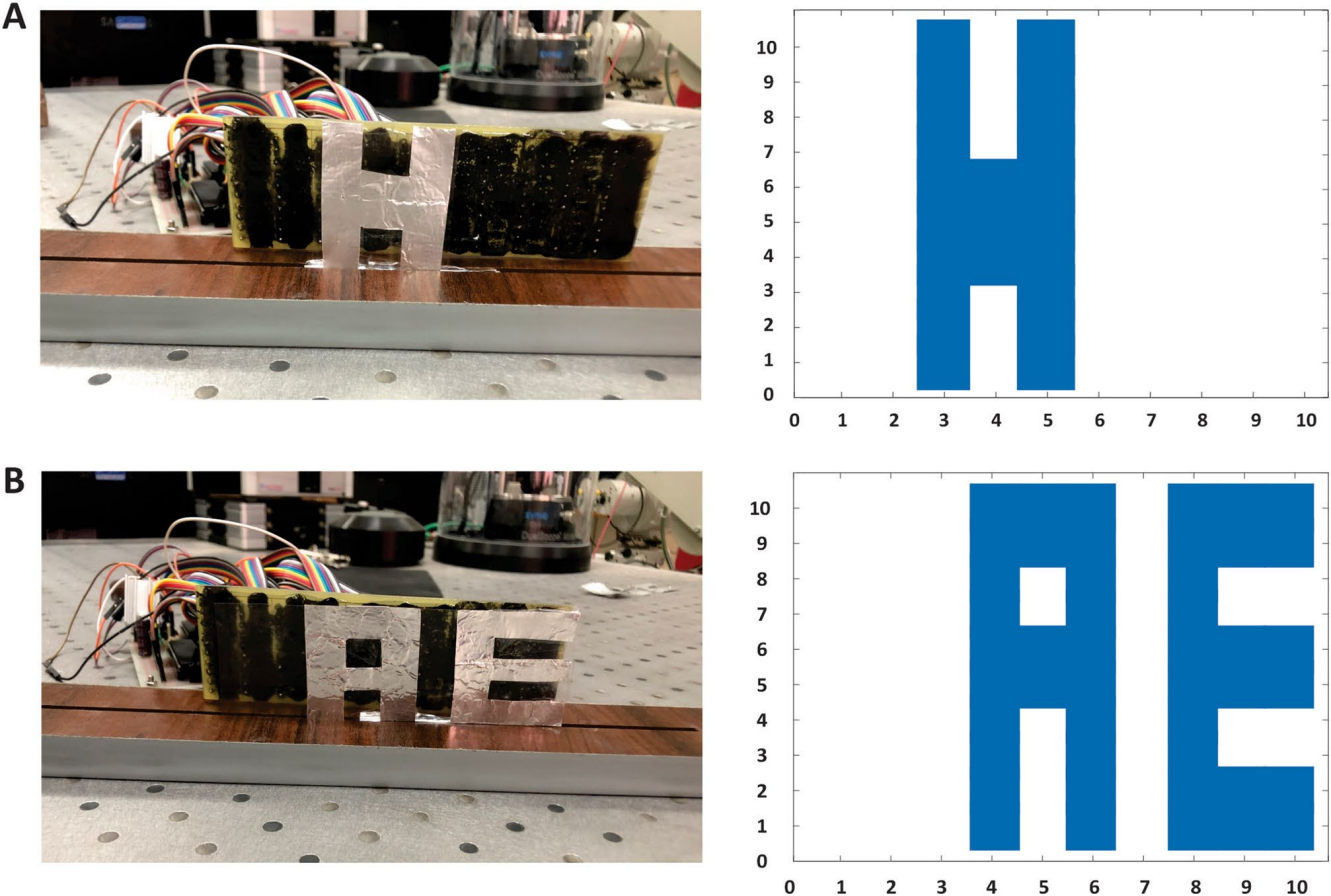
The image of different logos are constructed by the IR camera and displayed on the monitor. The images of the (**A**) H and (**B**) AE logos obtained by the proposed IR camera.

As we know, when the light source is radiated to the photodetector array structure, the resistance of those pixels which are exposed by the light will be decreased because of an increase in the carrier density and conductivity of their material. Due to this fact, we provided a logo in oic shape and placed it on the photodetector array structure. First, in dark conditions (without any IR radiation), the resistances are calculated and saved. Then by exposing IR light source and measuring the variation of the resistances of the sensors, the intensity of IR light can be detected. Consequently, with processing the received data, a figure of the oic logo will be displayed on the monitor. A real image of the fabricated device is depicted in Fig. 6B. Furthermore, as shown in Fig. 7, the images of different logos are constructed by the camera and displayed on the monitor.

**Figure 8:**
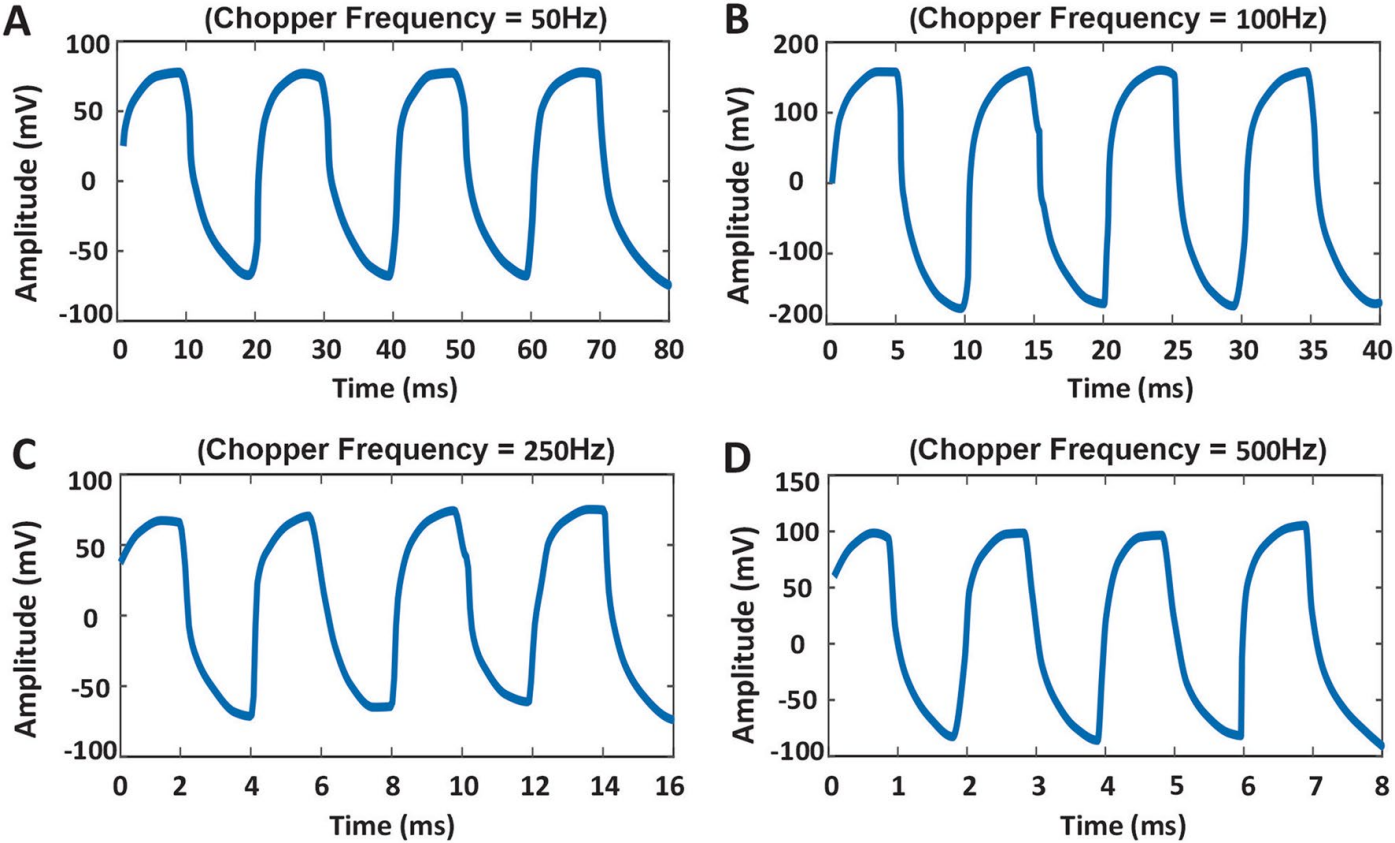
Response time of the fabricated IR camera measured practically. Response time for frequencies: (**A**) *f* = 50 Hz, rise time = 3.03 ms, (**B**) *f* = 100 Hz, rise time = 1.98 ms, (**C**) *f* = 250 Hz, rise time = 0.73 ms and (**D**) *f* = 500 Hz, rise time = 0.363 ms.

Besides, the frequency response of the designed device is practically measured to obtain the IR camera speed. Due to the device is fabricated for laboratory testing, the microprocessor, which has been used in the mentioned electronic circuit, caused a reduction in imaging speed. So, for calculating the speed of the camera, the device’s response time and fall time are measured for just one pixel of the photodetector array structure and plotted for four different frequencies of 50 Hz*,* 100 Hz, 250 Hz, and 500 Hz as shown in Fig. 8.

**Figure 9:**
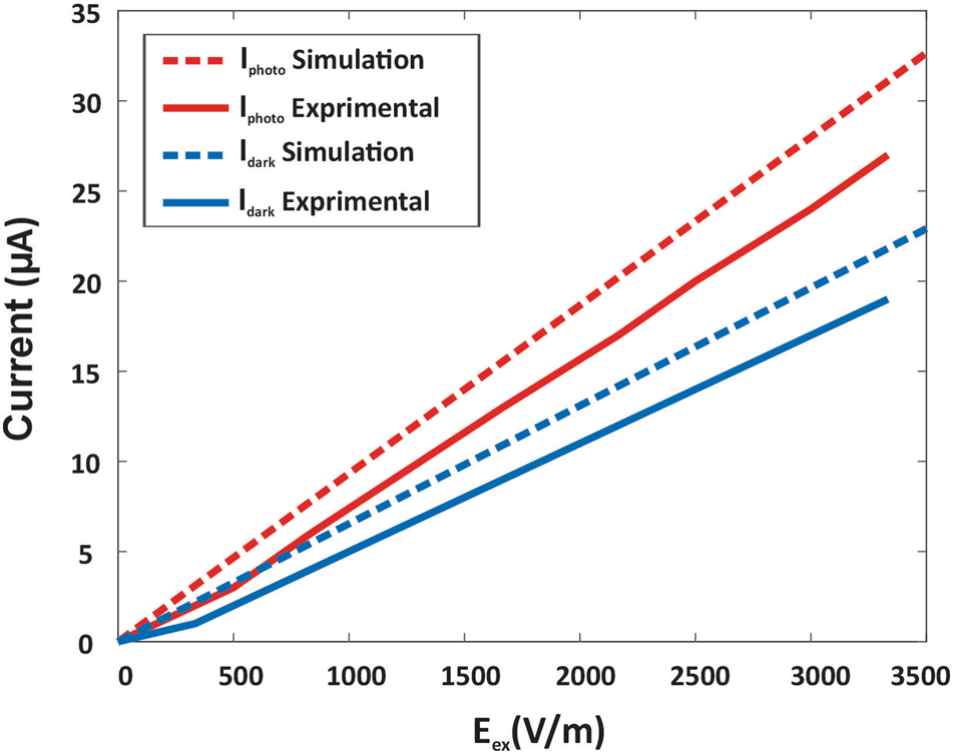
Comparison of simulation and experimental results by measuring the currents for fabricated IR camera. Dark currents and Photocurrents of the theoretical and experimental measurements.

Also, the dark current and photocurrent of the proposed device are measured practically for one pixel of the IR camera and compared with simulation results. Therefore, as indicated in Fig. 9, theoretical and experimental results are consistent with a little difference arising from ambient noise and signal-processing device delays.

## Conclusion

Finally, in this paper, a mid-infrared (3–5 µm) night vision high-speed camera based on solution-processed PbSe/PbI_2_ quantum dot photodetectors has been designed and fabricated. The electronic system to read data and process those to make image was implemented. The imaging mechanism of the proposed camera is based on the variation of conductivity due to applied IR light. The conductivity of the pixels with absorbing IR light is enhanced, and according to changes in conductivity, the image of an object is reconstructed. Optical and electrical properties were evaluated and measured. The proposed camera provides broadband operation, short response time, high responsivity, and detectivity. Also, the introduced camera offers some advantages such as simplicity, low-cost fabrication condition, high-performance, high speed, and working at room temperature.
